# Phenotypic Characteristics of B-Cell Subsets in Children with Allergic Diseases

**DOI:** 10.3390/ijms27198600

**Published:** 2026-09-25

**Authors:** Agnieszka Lipińska-Opałka, Agata Tomaszewska, Monika Leśnik, Robert Zdanowski, Michalina Leszczyńska-Pilich, Bolesław Kalicki

**Affiliations:** 1 Faculty of Medicine, University of Warsaw, 02-089 Warsaw, Poland; awawrzyniak@wim.mil.pl (A.T.); b.kalicki@uw.edu.pl (B.K.); 2 Department of Pediatrics, Nephrology and Allergology, Military Institute of Medicine–National Research Institute, 01-141 Warsaw, Poland; mleszczynska@wim.mil.pl; 3 Laboratory of Molecular Oncology and Innovative Therapies, Military Institute of Medicine National Research Institute, 04-141 Warsaw, Poland; mlesniak@wim.mil.pl (M.L.); rzdanowski@wim.mil.pl (R.Z.)

**Keywords:** B-cell subsets, allergic diseases, asthma, atopic dermatitis, allergic rhinitis, children

## Abstract

B-cell subpopulations are increasingly recognized as important modulators of immune responses, yet their role in pediatric allergic diseases remains incompletely understood. This study aimed to characterize the phenotype and frequency of peripheral B-cell subsets in children with allergic diseases and to assess their relationship with clinical and laboratory parameters. The study included 59 children with asthma, atopic dermatitis, or allergic rhinitis and 19 healthy controls. Multicolor flow cytometry was used to analyze B-cell subpopulations, including CD19^+^CD24hiCD38hi, CD19^+^CD25^+^, and IgD^−^CD27^+^ cells. The children with allergic diseases exhibited significantly reduced percentages of CD19^+^CD25^+^ B cells and switched-memory B cells compared with the controls. Negative correlations were observed between selected B-cell subsets and eosinophil counts, suggesting an association with type 2 inflammation. In asthma, higher proportions of transitional B cells were associated with better disease control, whereas no significant relationship was found between B-cell subsets and atopic dermatitis severity. In allergic rhinitis, the frequency of CD19^+^CD25^+^FoxP3^+^ B cells differed according to disease control, with lower frequencies observed in children with uncontrolled compared with partially controlled disease. These findings indicate that changes in B-cell populations may be involved in the pathogenesis of allergic diseases in children and their clinical expression.

## 1. Introduction

Over recent decades, the prevalence of allergic diseases, including asthma, atopic dermatitis (AD), and allergic rhinitis, has markedly increased in the pediatric population. While the pathophysiology of these diseases is complex and slightly different for individual disease entities, the effector cells involved in the allergic inflammatory process are often similar.

The first and best-studied cells involved in this process are Th2 lymphocytes, which promote the development of type 2 inflammation by secreting cytokines such as IL-4, IL-5, IL-13, and IL-31 [1].

In atopic dermatitis, the type 2 immune response plays a key role in the pathogenesis of the disease. Cytokines produced by Th2 lymphocytes contribute to epidermal barrier dysfunction, including reduced expression of filaggrin and antimicrobial peptides, even in the absence of primary genetic defects affecting the skin barrier. This highlights the close interdependence of two theories of atopic dermatitis pathogenesis, known as the inside-out and outside-in hypotheses. The crucial role of T lymphocytes in AD is supported by the clinical efficacy of T cell-targeted therapies and monoclonal antibodies directed against key Th2 cytokines. Furthermore, AD is maintained by a chronic itch-scratch cycle because sensory neurons also express receptors for Th2-dependent cytokines. Thus, cytokines such as IL-4, IL-13, and IL-31 contribute not only to skin inflammation but also to neuronal sensitization, thereby inducing the characteristic itch of the disease [2].

Similarly, in bronchial asthma and allergic rhinitis—despite the location of the inflammatory process in other organs—the main effector role is played by Th2 lymphocytes and the cytokines they produce (including IL-4, IL-5 and IL-13), which drive further stages of the inflammatory cascade, including the activation of B lymphocytes and their production of IgE antibodies [3].

However, Th2 lymphocytes are not the only culprits of inflammation in allergic diseases, as evidenced by the fact that drugs directed against these cells and their cytokines are not effective in all patients. T-helper type 1 (Th1) and type 17 (Th17) lymphocyte responses may also be increased in allergic diseases, particularly in chronic lesions (Th1) [3]. Regulatory T cells are well known for their role in suppressing the excessive immune response seen in allergic diseases [4]. However, much less is known about the role of B-cell subsets in this process [5].

B-cell subpopulations are a heterogeneous population capable of suppressing immune responses via the secretion of anti-inflammatory cytokines—most notably IL-10 and IL-35—or by direct interaction with T cells. Their role in maintaining peripheral tolerance has been demonstrated in various autoimmune and allergic models [5,6]. Impaired B-cell number or function has been linked to increased disease severity in asthma, atopic dermatitis, and food allergies [7,8].

In our previous review, we summarized evidence that B-cell subsets are decreased in several allergic conditions and their functional capacity—particularly IL-10 production—is often diminished [9]. However, pediatric data remain limited and fragmented. Furthermore, few studies have analyzed the relationship between B-cell subsets and clinical features such as eosinophilia or asthma control.

The present study aimed to characterize the phenotype and frequency of B-cell subsets and B regs in peripheral blood of children with allergic diseases and to assess their correlation with clinical parameters and laboratory markers of inflammation.

## 2. Results

### 2.1. Characteristics of the Group

The study included 59 children in the study group (median age of 5 years; q25 = 3.0, q75 = 8.0) and 19 children in the control group (median age of 7 years; q25 = 4.5, q75 = 8.0). The groups did not differ in terms of gender distribution (*p* ^chi2^ = 0.102). Nevertheless, statistically significantly higher percentages of eosinophils in peripheral blood and concentrations of total IgE antibodies were observed in the study group compared with the control group (p^UMW^ = 0.002 and p^UMW^ < 0.001, respectively) (Table 1).

The most common allergic disease was allergic rhinitis (n = 41, 53%). Asthma and atopic dermatitis were diagnosed in 30 (38%) and 37 (47%) children, respectively. Asthma and allergic rhinitis coexisted in 27 participants, representing 90% of the children with asthma. Figure 1 presents the distribution of disease control or severity categories in the study group.

### 2.2. B-Cell Subsets in Allergic Diseases

In the first stage of the analysis, the percentage values of lymphocytes were compared between the study group and the control group. To better differentiate between B-lymphocyte subclasses, CD19+ cells were initially selected, and then surface antigens (CD38, CD24, CD27, and IgD) were used. Statistically significantly lower values of lymphocytes with the IgD-CD27+SM phenotype were observed in the study group compared with the control group (*p* = 0.035) (Table 2, Figure 2).

In the group of children with allergies, a statistically significantly lower percentage of CD19+CD25+ lymphocytes was observed compared with the children from the control group (15.44% ± 6.86 and 21.41% ± 6.73, respectively; *p* < 0.001). B cells expressing the FoxP3 molecule were identified in peripheral blood of both the study group and the control group. They were found to constitute 1.20% (IQR: 0.26–2.46) of CD19+CD25+ cells in the study group and 1.60% (IQR: 0.87–3.14) of CD19+CD25+ cells in the control group. However, the difference in percentage values was not statistically significant (*p* = 0.126). On the other hand, lower percentage values of CD19+CD25+ lymphocytes not expressing the FoxP3 molecule were observed in the study group (*p* < 0.001) (Figure 3).

When analyzing the correlations between the concentration of total IgE antibodies, the absolute values of eosinophils, and the assessed populations of B-cell subsets, a statistically significant negative linear relationship was observed in the entire study group between lymphocytes with the CD19+CD38+CD24+ phenotype and eosinophilia (Pearson’s correlation coefficient r = (−)0.269; *p* = 0.018). After considering the division into the study and control groups, the observed relationship was present only in the study group (Pearson’s correlation coefficient r = (−)0.313; *p* = 0.016). In the group of children with allergies, a negative correlation was also observed between CD19+CD38+CD24− lymphocytes and the eosinophilia peripheral blood (Pearson’s correlation coefficient r = (−)0.284; *p* = 0.030) (Figure 4).

### 2.3. B-Cell Subsets and the Severity of Allergic Diseases

#### 2.3.1. Asthma

In the group of children with asthma, complete disease control was observed in 6 children, partial control was observed in 18 patients, and uncontrolled asthma was observed in 6 children. The distribution of percentage values of lymphocytes with the CD38hiCd24hi TRANS phenotype differed statistically between the analyzed groups (*p* = 0.029) (Table 3). Post hoc analysis showed significantly higher CD38hiCd24hi TRANS values in the group of children with controlled asthma compared with the group of children with uncontrolled asthma (*p* = 0.008).

#### 2.3.2. Atopic Dermatitis

In the group of children with atopic dermatitis, mild severity of the disease was found in 19 children, moderate severity was found in 14 patients, and severe severity was found in 4 children.

No statistically significant differences in lymphocyte percentages were observed depending on the severity of atopic dermatitis (Table 4).

#### 2.3.3. Allergic Rhinitis

Among children with allergic rhinitis, the disease was completely controlled in 26 patients, partially controlled in 8 patients, and uncontrolled in 7 patients. The distribution of CD19^+^CD25^+^FoxP3^+^ B-cell frequencies differed significantly across the three disease control groups (Kruskal–Wallis *p* = 0.043) (Table 5). Post hoc analysis showed significantly lower frequencies of CD19^+^CD25^+^FoxP3^+^ B cells in children with uncontrolled allergic rhinitis compared with those with partially controlled disease (median: 0.64 versus 2.35, respectively; *p* = 0.015). The difference between children with completely controlled and uncontrolled allergic rhinitis was of borderline statistical significance (median: 1.34 versus 0.64, respectively; *p* = 0.054).

## 3. Discussion

### 3.1. B-Cell Subsets in Allergy

Regulatory B cells (Bregs) represent a specialized subset of B lymphocytes with important immunomodulatory properties. Their regulatory activity is largely mediated by the secretion of anti-inflammatory cytokines, including interleukin-10 (IL-10), inter-leukin-35 (IL-35), and transforming growth factor-β (TGF-β). Although several phenotypic markers associated with B cells have been proposed, their definitive identification in allergic diseases remains challenging. In humans, B-cell populations include, among others, CD19^+^CD24hiCD38hi B cells, CD19^+^CD24hiCD27^+^ B cells, and CD19^+^CD25hiCD71hiCD73^−^ B cells [10]. Other studies have reported additional phenotypic characteristics of regulatory B cells, indicating that B cells producing IL-10 and TGF-β may also exhibit high expression of FoxP3 and CD5. This observation suggests the presence of another regulatory subset characterized by a CD19^+^CD5highFoxP3highIL-10high phenotype. These cells have been implicated in the modulation of inflammatory responses and have been described in the context of disorders such as non–IgE-mediated food allergies and atopic dermatitis [11].

Our findings demonstrate that children with allergic diseases exhibit significant alterations in the phenotype of peripheral B-cell subsets. Most notably, a reduction in CD19^+^CD25^+^ and switched memory B-cell (IgD^−^CD27^+^) populations was observed in the allergic children compared with the healthy controls.

This aligns with previous studies suggesting that B cells, particularly IL-10-producing subsets, are diminished in allergic conditions. A group of Chinese scientists found a lower percentage of lymphocytes expressing CD24, CD27, and CD38 molecules in patients with atopic dermatitis compared with healthy individuals. They also demonstrated that B lymphocytes expressing these molecules produced Il-10 [12]. Moreover, Kamekura et al. reported a significant reduction in the frequency of CD19^+^CD24hiCD27^+^ B cells in patients with allergic rhinitis and asthma [13]. Similarly, Sheehan et al. observed decreased circulating CD24^+^CD38^+^ B-cell levels in children with asthma compared with healthy controls [14].

Furthermore, an expansion of peripheral CD19^+^CD25^+^CD71^+^CD73^−^ B cells has been observed during allergen-specific immunotherapy, and it was associated with decreased IgE levels, enhanced IgG4 production, and increased IL-10 secretion [15]. These findings suggest that changes in specific B-cell populations may accompany the development of immune tolerance during allergen-specific immunotherapy. In this context, the reduced frequency of CD19^+^CD25^+^ B cells observed in our cohort of children with allergic diseases may reflect alterations in B-cell populations associated with ongoing allergic inflammation. However, the functional significance of this finding cannot be determined from our phenotypic analysis alone.

### 3.2. Severity of Allergic Diseases

This imbalance in B-cell subpopulations may therefore contribute to the persistence or exacerbation of allergic disease. Building on this observation, we further analyzed the relationship between B-cell subsets and the clinical severity of individual allergic diseases in our cohort. In the asthma subgroup, children with controlled disease exhibited higher percentages of CD38hiCD24hi transitional B cells compared with those who presented uncontrolled asthma. This finding is consistent with the concept that transitional B-cell populations contribute to the maintenance of immune homeostasis and may be associated with improved control of airway inflammation. Previous studies suggested that a reduced number of IL-10-producing B-cell subpopulations might be associated with more severe forms of allergic airway disease [7].

By contrast, in children with atopic dermatitis, we did not observe significant differences in the proportions of the analyzed B-cell populations according to disease severity. Previous studies have reported both quantitative and functional alterations in select B-cell subsets in patients with atopic dermatitis. Jiang et al. found that children with moderate-to-severe atopic dermatitis had a lower proportion of IL-10-producing CD19+ B cells than those with mild disease. However, when all patients with atopic dermatitis were analyzed together, the percentage of IL-10-producing CD19+ B cells did not correlate significantly with SCORAD. After stratification according to atopic dermatitis phenotype, a significant negative correlation between the frequency of IL-10-producing CD19+ B cells and SCORAD was observed specifically in patients with extrinsic atopic dermatitis (EAD) [8]. The same study also demonstrated impaired suppressive activity of CD19+CD24^hi^CD38^hi^ B cells from patients with EAD in an in vitro Tfh cell differentiation assay, indicating that functional alterations may occur even when differences in the frequencies of phenotypically defined B-cell subsets are not apparent. In our cohort, we found no significant association between the frequencies of the analyzed B-cell populations and atopic dermatitis severity. This discrepancy may partly reflect the lack of stratification into intrinsic and extrinsic forms of atopic dermatitis in our study, as well as the clinical and immunological heterogeneity of the disease and the relatively small number of patients with severe atopic dermatitis. Moreover, our analysis was based on the frequencies of phenotypically defined B-cell subsets and did not directly assess their functional properties.

In allergic rhinitis, the relationship between CD19^+^CD25^+^FoxP3^+^ B-cell frequencies and disease control appeared more complex. We observed significantly lower frequencies of CD19^+^CD25^+^FoxP3^+^ B cells in children with uncontrolled allergic rhinitis compared with those with partially controlled disease. A borderline difference was also observed between the children with completely controlled and uncontrolled allergic rhinitis. These findings suggest that poorer disease control may be associated with reduced frequencies of this phenotypically defined B-cell population. However, the nonlinear distribution across the three disease control groups indicates that this relationship should be interpreted cautiously and requires confirmation in larger cohorts.

Previous studies have described both quantitative and functional alterations of B-cell subsets in allergic rhinitis, including reduced frequencies of CD19^+^CD25^+^ B cells and other B-cell populations associated with immunoregulatory properties [9,16]. Our findings are therefore broadly consistent with the concept that alterations in select B-cell subsets may accompany more active or poorly controlled allergic inflammation.

Importantly, the CD19^+^CD25^+^FoxP3^+^ phenotype investigated in our study does not correspond to a classical or widely validated human B-cell subset with established immunoregulatory function [17,18]. Although CD25 expression has been associated with immunoregulatory properties in B lymphocytes, the significance of FoxP3 expression in B cells remains controversial, and FoxP3 is not routinely used as a defining marker of immunoregulatory B-cell populations. FoxP3 expression in B cells was first reported in 2010 [19], and subsequent studies have investigated CD19^+^CD25^+^FoxP3^+^ B cells in the context of food allergies. In one such study, milk exposure resulted in a decrease in the proportion of CD19^+^CD25^+^FoxP3^+^ B cells in patients with a casein allergy, whereas an increase in this population was observed in healthy individuals under the same conditions [20].

Data on the association between CD19^+^CD25^+^FoxP3^+^ B-cell frequencies and allergic rhinitis control remain limited. Therefore, our findings should be regarded as exploratory and hypothesis-generating. They indicate an association between disease control and the frequency of this phenotypically defined B-cell population but do not establish its regulatory function or a causal role in allergic rhinitis.

### 3.3. Eosinophil, IgE

The significant negative correlations observed between B-cell subsets and peripheral eosinophil counts and suggest a close relationship between B-cell subset insufficiency and type 2 inflammatory activity. These findings are consistent with the previous literature linking decreased IL-10-producing B cells to elevated Th2 activity, eosinophilia, and poor disease control [9]. Experimental data indicate that IL-10–producing B cells directly suppress eosinophil activation and migration [21,22], while their deficiency correlates with enhanced eosinophilia and Th2 polarization across multiple allergic conditions [23,24]. This inverse relationship implies that regulatory B cells may play a role in modulating eosinophil-associated inflammation.

### 3.4. Limitations

This study has several strengths. These include comprehensive immunophenotyping of B-cell subsets using an expanded flow cytometry panel and correlation of immuno-logical test results with clinical variables such as asthma control and eosinophilia. However, certain limitations should be noted. Given the large number of B-cell subsets, disease groups, and clinical correlations evaluated, no correction for multiple testing was applied across the study as a whole; post hoc Bonferroni correction was limited to pairwise comparisons within each individual Kruskal–Wallis analysis. This approach reflects the exploratory character of the study and increases the risk of false-positive findings. Consequently, the reported associations should be interpreted as hypothesis-generating rather than confirmatory, and future studies with pre-specified primary endpoints and appropriate correction for multiplicity are warranted to validate these findings. The sample size in the control group was relatively small, and the cross-sectional nature of the study limits causal interpretation. It is worth emphasizing, however, that this study is ongoing, and its next phase will include comparing the activity of cytokines produced by regulatory B cells between individual patient groups and according to disease severity.

## 4. Materials and Methods

### 4.1. Study Cohort

The study cohort included 59 children with allergic diseases and 19 age- and sex-matched healthy controls. The study group included patients aged 3–8 years with a diagnosis of at least one of the following allergic diseases: asthma, allergic rhinitis, or atopic dermatitis. Exclusion criteria were the coexistence of immunological disorders, other chronic skin diseases, or respiratory diseases and lack of informed consent.

During the physical examination, the current severity or level of exacerbation of the allergic disease was assessed. For this purpose, the following validated instruments were used: the SCORing Atopic Dermatitis (SCORAD) index for atopic dermatitis, the Asthma Control Test (ACT) for asthma, and the visual analogue scale (VAS) for allergic rhinitis. Asthma control was classified according to the Global Initiative for Asthma (GINA) guidelines as controlled, partially controlled, and uncontrolled. The severity of atopic dermatitis was assessed using the SCORing Atopic Dermatitis (SCORAD) index and categorized as mild, moderate, or severe according to established cut-off values. Allergic rhinitis control was assessed using a 10-cm visual analogue scale (VAS) and categorized as completely controlled (VAS of 0–2), partially controlled (VAS > 2–5), or uncontrolled (VAS > 5–10). These categories were used consistently to stratify patients for the disease-specific analyses.

The study was conducted in accordance with the principles of the Declaration of Helsinki. The study protocol was reviewed and approved by the Bioethics Committee at the Military Medical Chamber (approval no. 218/22). Written informed consent was obtained from the parents or legal guardians of all participating children prior to enrollment.

### 4.2. Immunophenotyping of Peripheral Blood Cell

Peripheral venous blood was collected into BD Vacutainer tubes (Cat. No. 367836, BD, Wokingham, UK) containing K_2_EDTA as an anticoagulant and used for immunophenotyping. Surface and intracellular staining was performed using the fluorochrome-conjugated antibodies listed in Table 6. Intracellular staining of FoxP3 antigen was carried out using the PerFix-NC kit (Cat. No. B31168, Beckman Coulter, Brea, CA, USA) according to the manufacturer’s protocols. Samples were acquired on a CytoFLEX flow cytometer (Beckman Coulter, USA; 2019) and analyzed using CytExpert software (version 2.3.0.84, Beckman Coulter, USA).

To ensure measurement reproducibility and verify the optimal performance of the CytoFLEX flow cytometer (Beckman Coulter, USA), instrument quality control was performed prior to each acquisition session using CytoFLEX Ready to Use Daily QC Fluorospheres (C65719, Beckman Coulter, USA) according to the manufacturer’s instructions. For multicolor flow cytometry analyses, fluorescence compensation was performed to correct for spectral overlap between individual fluorescence detection channels. Compensation controls were prepared using the VersaComp Antibody Capture Bead Kit (B22804, Beckman Coulter, USA) in accordance with the manufacturer’s recommendations. Compensation settings were established prior to sample analysis and applied to the corresponding multicolor staining panels.

During flow cytometric acquisition, 30,000 events within the CD45^+^ LYMPH gate were collected for each sample (Figure 5A). These CD45^+^ events constituted the parent population for subsequent identification and analysis of the investigated B-cell subsets.

### 4.3. Gating Strategy

The total lymphocyte population (LYMPH) was identified based on side scatter (SSC-A) characteristics and CD45 expression (Figure 5A). Next, B lymphocytes were isolated from the LYMPH population based on the co-expression of CD45 and CD19 (Figure 5B). Within the CD45^+^CD19^+^ B-cell population, individual B-cell subpopulations were identified based on the expression of IgD, CD27, CD24, CD38, CD25, and FoxP3. Based on the expression of IgD and CD27, B cells were classified as non-switched-memory B cells (IgD^+^CD27^+^; nSM), naïve B cells (IgD^+^CD27^−^; Naïve), switched-memory B cells (IgD^−^CD27^+^; SM), and double-negative B cells (IgD^−^CD27^−^; DN) (Figure 5C). Transitional B cells were defined as D38^Hi^CD24^Hi^ cells, while plasmablasts were identified as CD38^Hi^CD24^Low^ cells (Figure 5D). Furthermore, the CD19^+^ B cells were further subdivided into CD25^+^ and CD25^−^ subpopulations based on CD25 expression (Figure 5E). The subpopulation of CD19^+^CD25^+/−^FoxP3^+/−^ B cells was identified directly within the parent population of CD45^+^CD19^+^ B cells based on the co-expression of CD25 and intracellular FoxP3 (Figure 5F) [25,26].

### 4.4. Laboratory Parameters

Peripheral venous blood samples were collected for routine laboratory analyses. Complete blood cell counts, including absolute and relative eosinophil counts, were determined using an automated hematology analyzer. Serum C-reactive protein (CRP) concentrations were measured using a standard immunoturbidimetric method. The total serum IgE concentrations were determined using the ImmunoCAP system (Thermo Fisher Scientific, Uppsala, Sweden). All laboratory measurements were performed according to the manufacturers’ instructions and routine procedures of the hospital diagnostic laboratory.

### 4.5. Statistical Analyses

Statistical analyses were performed using SPSS software version 26.0 (IBM Corp., Armonk, NY, USA). Data normality was assessed with the Shapiro–Wilk test. Depending on the data distribution, comparisons between two groups were conducted using the Mann–Whitney U test or Student’s *t*-test, while the Kruskal–Wallis test was applied for multiple group comparisons. Following significant Kruskal–Wallis results, post hoc pairwise comparisons were performed using the Mann–Whitney U test with Bonferroni correction within each family of comparisons. No correction for multiple testing was applied across the different B-cell subsets, disease groups, and correlation analyses evaluated (see Limitations). Associations between variables were evaluated using Pearson’s or Spearman’s correlation coefficients, as appropriate. A *p* value <0.05 was considered statistically significant.

## 5. Conclusions

Our study demonstrated alterations in selected peripheral B-cell populations in children with allergic diseases, particularly reduced frequencies of CD19^+^CD25^+^ and switched-memory B cells compared with healthy controls. Associations between selected the B-cell subsets and markers of type 2 inflammation, including peripheral eosinophil counts and serum IgE levels, were also observed.

Exploratory disease-specific analyses revealed associations between the selected B-cell populations and measures of disease control or severity. In asthma, higher frequencies of transitional B cells were associated with better disease control, whereas no clear association with disease severity was observed in atopic dermatitis. These findings demonstrate alterations in the selected peripheral B-cell subsets in children with allergic diseases and indicate associations between their frequencies, type 2 inflammatory markers, and clinical disease control.

Overall, these findings indicate that alterations in peripheral B-cell phenotypes are associated with allergic inflammation and clinical disease characteristics in children. Given the cross-sectional design, small clinical subgroups, overlap between allergic diagnoses, and absence of direct functional assessment, these findings should be considered exploratory and hypothesis-generating. Further studies in larger pediatric cohorts incorporating functional analyses are needed to determine the biological significance of these B-cell populations in allergic diseases.

## Figures and Tables

**Figure 1:**
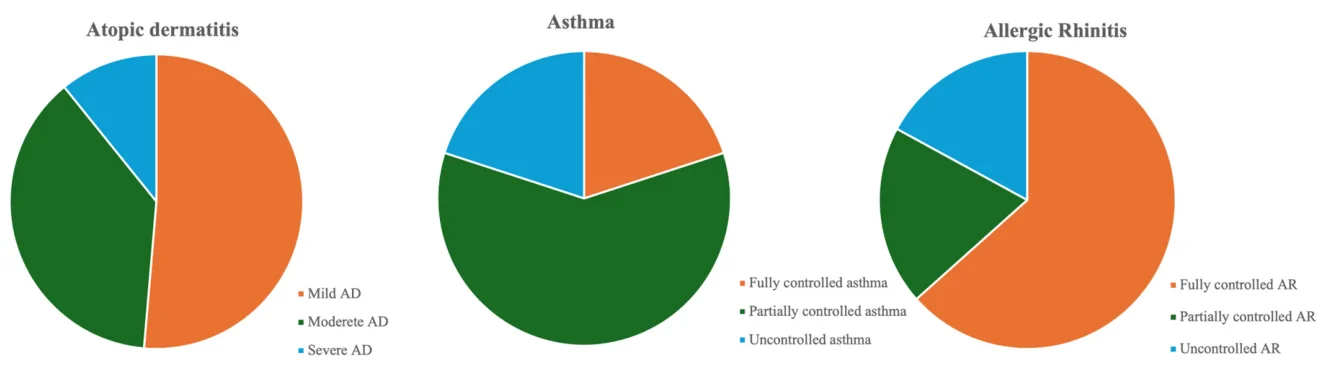
Distribution of disease control or severity categories in the study group.

**Figure 2:**
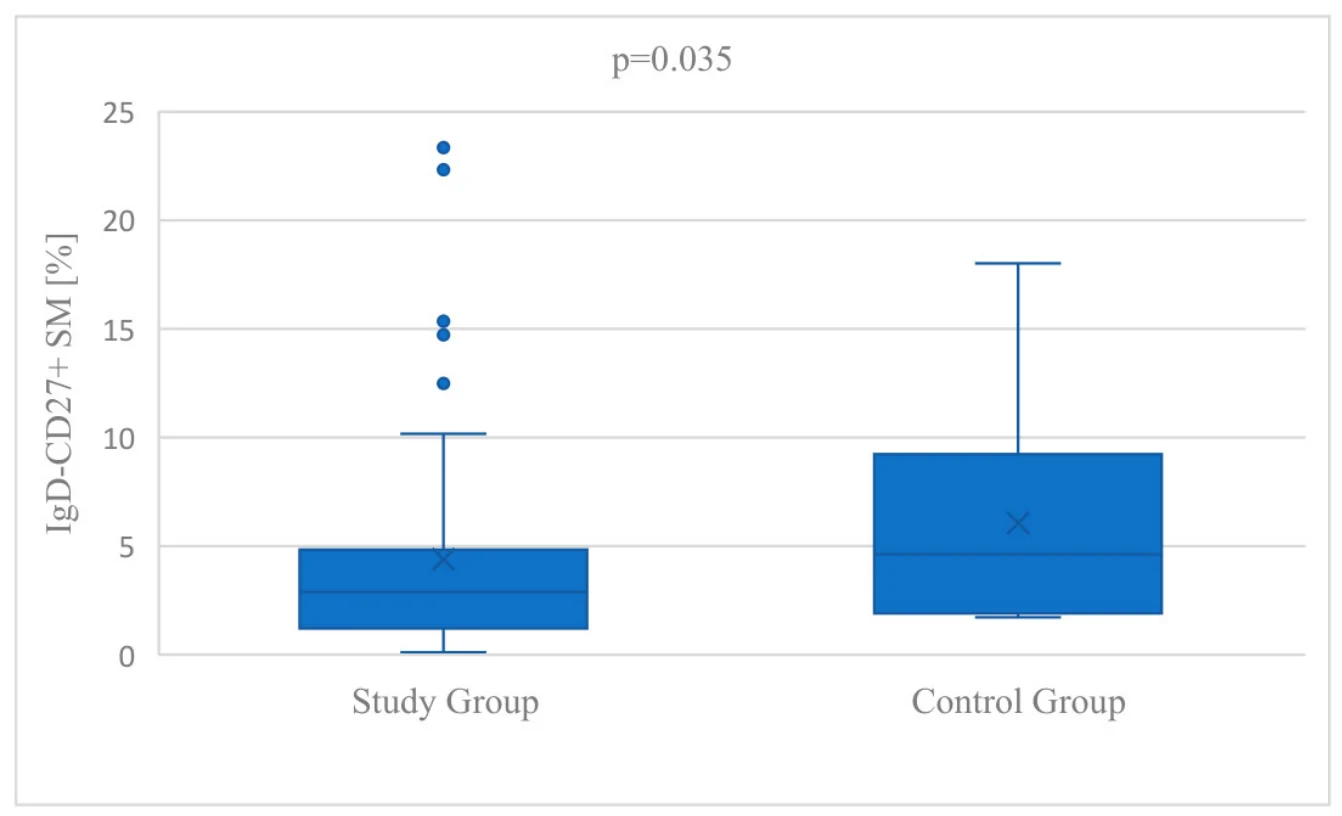
Percentage values of IgD-CD27+ lymphocytes in the study group and the control group (SM = switched memory).

**Figure 3:**
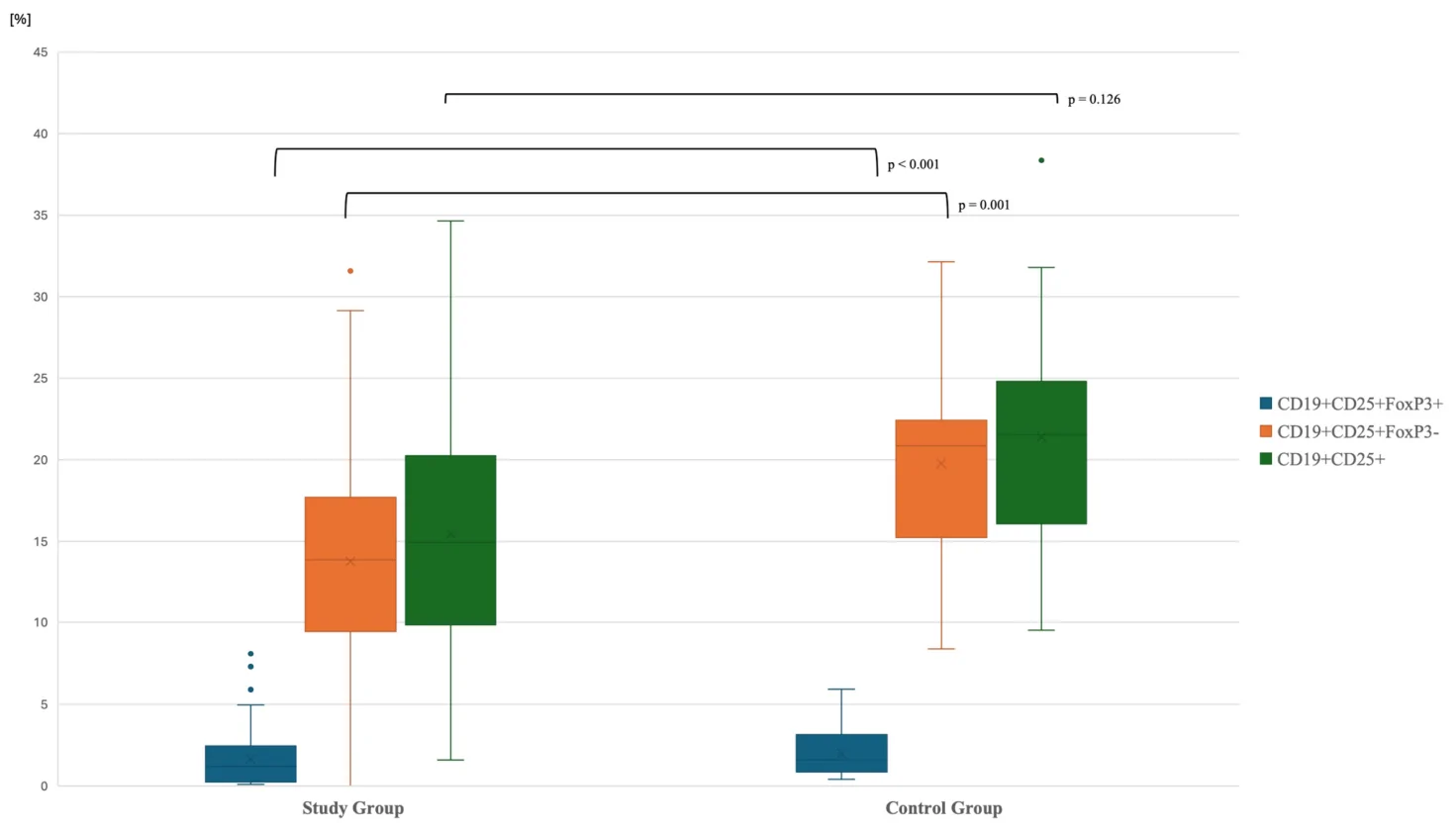
Percentage values of CD19+CD25+ lymphocytes (with and without FoxP3 molecule expression) in the study group and in the control group.

**Figure 4:**
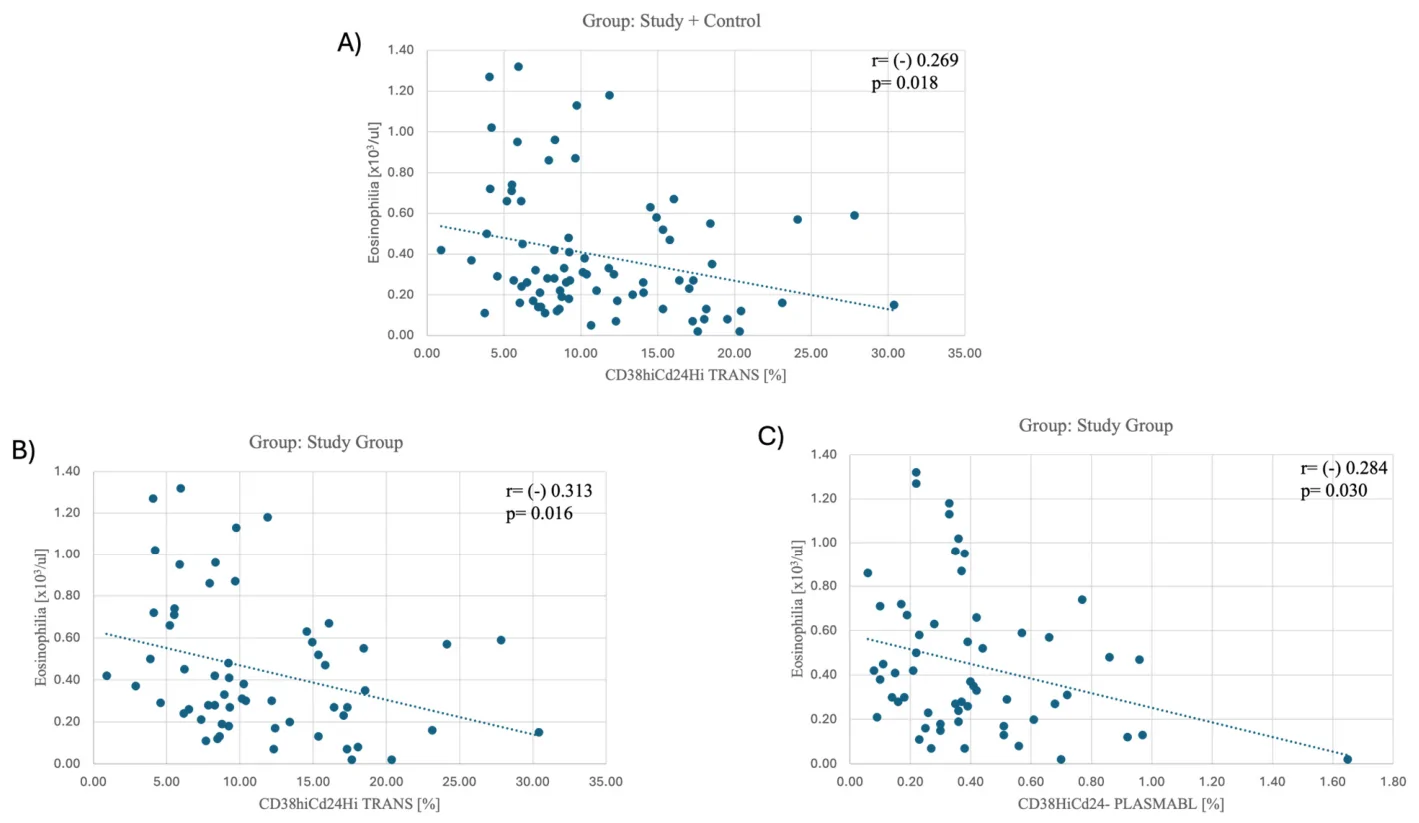
Correlation between eosinophilia and the percentage of CD19+CD38+CD24+ lymphocytes in the entire analyzed group (**A**) and correlations between eosinophilia and the percentage of CD19+CD38+CD24+ (**B**) and CD19+CD38+CD24− (**C**) lymphocytes in the study group.

**Figure 5:**
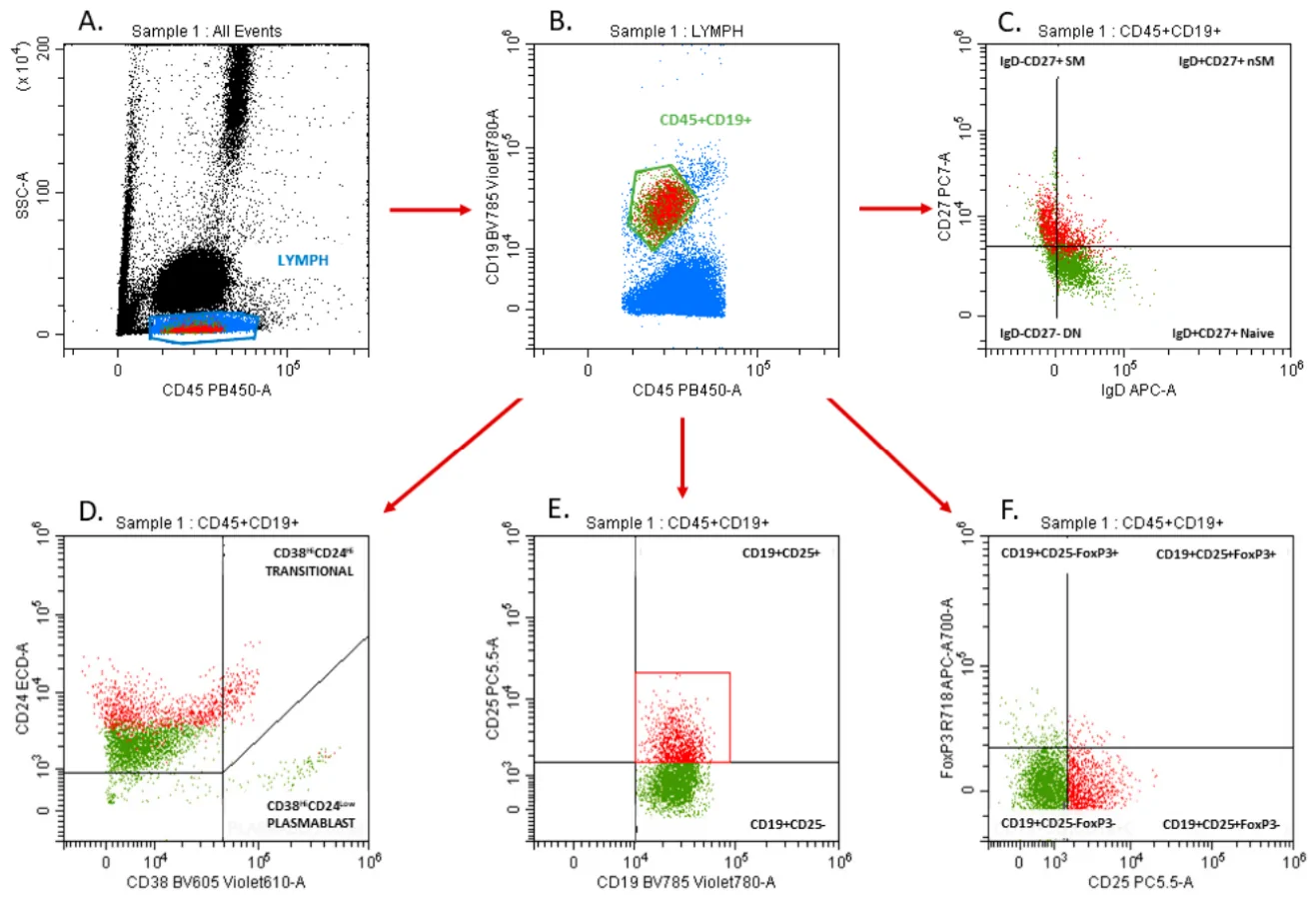
Gating strategy used to determination of B-cell subsets: (**A**) population of total lymphocytes, (**B**) population of B lymphocytes, (**C**) naïve, non-switched-memory, switched-memory, and double-negative B lymphocytes, (**D**) transitional B lymphocytes and plasmablasts, (**E**) B lymphocytes with expression CD19^+^CD25^+/−^, and (**F**) B lymphocytes with expression of CD19^+^CD25^+/−^FoxP3^+/−^. B-cell subsets are presented as a percentage of the CD45^+^CD19^+^ parent population.

**Table 1:** Characteristics of the study group and the control group.

Variable	Study Group (n = 59)	Control Group (n = 19)	*p*
Age [years], median (IQR)	5.0 (3.0–8.0)	7.0 (4.5–8.0)	*p*^UMW^ = 0.172
Sex			
Boys; n (%)	41 (70%)	9 (47%)	*p*^chi2^ = 0.102
Girls; n (%)	18 (30%)	10 (53%)	
WBC [×10^9^/L], median (IQR)	7.92 (6.67–10.30)	5.82 (5.37–8.21)	*p*^UMW^ = 0.008
CRP [mg/dL], median (IQR)	0.1 (0.10–0.10)	0.1 (0.10–0.10)	*p*^UMW^ = 0.604
IgE [IU/mL], median (IQR)	133.0 (36.25–748.75)	13.0 (21.40–35.05)	*p*^UMW^ < 0.001
Eosinophils [%], median (IQR)	0.34 (0.20–0.65)	0.22 (0.15–0.29)	*p*^UMW^ = 0.002

*p* = statistical significance; WBC = white blood cell count; n = count; IQR = quartile range; UMW = U Mann–Whitney test; chi2 = test chi^2^.

**Table 2:** Percentage values of peripheral blood lymphocytes in the study group and the control group.

Variable	Study Group (n = 59)	Control Group (n = 19)	*p* Value
CD45^+^CD19^+^ [% of total], mean (±SD)	13.04 (±4.55)	12.82 (±3.99)	*p*^St^ = 0.851
CD38^hi^CD24^hi^ TRANS [% of CD45^+^CD19^+^], median (IQR)	9.29 (6.51–15.08)	9.04 (6.09–14.06)	*p*^UMW^ = 0.740
CD38^hi^CD24^Lo^ PLASMABL [% of CD45^+^CD19^+^], median (IQR)	0.36 (0.22–0.52)	0.26 (0.16–0.40)	*p*^UMW^ = 0.123
IgD^+^CD27^+^ nSM [% of CD45^+^CD19^+^], mean (±SD)	18.36 (±8.79)	16.60 (±8.98)	*p*^St^ = 0.453
IgD^−^CD27^+^ SM [% of CD45^+^CD19^+^], median (IQR)	2.89 (1.21–4.84)	4.63 (1.91–9.24)	*p*^UMW^ = 0.035
IgD^−^CD27^−^ DN [% of CD45^+^CD19^+^], median (IQR)	3.02 (1.68–4.24)	3.47 (1.51–4.22)	*p*^UMW^ = 0.811
IgD^+^CD27^−^ NAÏVE [% of CD45^+^CD19^+^], mean (±SD)	73.78 (±10.19)	74.35 (±9.88)	*p*^St^ = 0.834
CD19^+^CD25^+^ [% of CD45^+^CD19^+^], mean (±SD)	15.44 (±6.86)	21.41 (±6.73)	*p*^St^ < 0.001
CD19^+^CD25^+^FoxP3^+^ [% of CD45^+^CD19^+^], median (IQR)	1.20 (0.26–2.46)	1.60 (0.87–3.14)	*p*^UMW^ = 0.126
CD19^+^CD25^+^FoxP3^−^ [% of CD45^+^CD19^+^], mean (±SD)	13.72 (±6.26)	19.77 (±6.08)	*p*^St^ = 0.001

SD = standard deviation; IQR = interquartile range; St = Student’s *t*-test; UMW = U Mann–Whitney test; SM = switched memory; DN = double negative.

**Table 3:** Subpopulations of B-cell subsets in the group of children with controlled, partially controlled, and uncontrolled asthma.

Variable	Controlled Asthma (n = 6)	Partially Controlled Asthma (n = 18)	Uncontrolled Asthma (n = 6)	*p* Value
CD45^+^CD19^+^ [% of total], median, IQR	15.06 (10.97–17.40)	9.44 (7.21–11.93)	11.54 (6.93–16.47)	*p*^KW^ = 0.052
CD38^hi^Cd24^hi^ TRANS [% of CD45^+^CD19^+^], median, IQR	13.65 (9.16–16.01)	8.98 (5.28–10.94)	6.04 (3.79–8.47)	*p*^KW^ = 0.029
CD38^hi^Cd24^Lo^ PLASMABL [% of CD45^+^CD19^+^], median, IQR	0.33 (0.16–0.52)	0.35 (0.22–0.69)	0.36 (0.15–0.40)	*p*^KW^ = 0.600
IgD^+^CD27^+^ nSM [% of CD45^+^CD19^+^], median, IQR	20.16 (16.06–24.56)	18.59 (11.52–18.59)	19.56 (16.44–32.56)	*p*^KW^ = 0.734
IgD^−^CD27^+^ SM [% of CD45^+^CD19^+^], median, IQR	1.45 (0.99–8.20)	3.66 (1.59–8.64)	2.53 (0.72–5.12)	*p*^KW^ = 0.458
IgD^−^CD27^−^ DN [% of CD45^+^CD19^+^], median, IQR	2.95 (2.07–4.07)	4.11 (1.59–6.92)	2.57 (1.51–4.23)	*p*^KW^ = 0.556
IgD^+^CD27^−^ NAÏVE [% of CD45^+^CD19^+^], median, IQR	73.45 (65.77–78.76)	68.33 (63.04–75.67)	74.29 (60.37–78.26)	*p*^KW^ = 0.705
CD19^+^CD25^+^ [% of CD45^+^CD19^+^], median, IQR	13.90 (6.49–17.57)	14.93 (9.68–21.35)	16.79 (12.72–25.38)	*p*^KW^ = 0.308
CD19^+^CD25^+^FoxP3^+^ [% of CD45^+^CD19^+^], median, IQR	1.38 (0.31–2.39)	1.22 (0.74–1.88)	2.61 (1.22–3.79)	*p*^KW^ = 0.263
CD19^+^CD25^+^FoxP3^−^ [% of CD45^+^CD19^+^], median, IQR	11.79 (6.24–15.31)	13.88 (9.27–18.37)	13.40 (9.85–22.30)	*p*^KW^ = 0.149

IQR = interquartile range; KW = Kruskal–Wallis test; SM = switched memory; DN = double negative.

**Table 4:** Subpopulations of B-cell subsets in the group of children with mild, moderate, and severe atopic dermatitis.

Variable	Mild Atopic Dermatitis (n = 19)	Moderate Atopic Dermatitis (n = 14)	Severe Atopic Dermatitis (n = 4)	*p* Value
CD45^+^CD19^+^ [% of total]; median, IQR	14.58 (10.97–17.40)	13.28 (7.21–11.93)	13.13 (6.93–16.47)	*p*^KW^ = 0.742
CD38^hi^Cd24^hi^ TRANS [% of CD45^+^CD19^+^], median, IQR	12.38 (8.60–17.29)	10.39 (6.42–17.82)	8.10 (5.88–14.11)	*p*^KW^ = 0.364
CD38^hi^Cd24^Lo^ PLASMABL [% of CD45^+^CD19^+^], median, IQR	0.37 (0.26–0.66)	0.37 (0.21–0.60)	0.17 (0.08–0.36)	*p*^KW^ = 0.216
IgD^+^CD27^+^ nSM [%of CD45^+^CD19^+^], median, IQR	12.37 (9.73–22.34)	20.38 (10.49–30.16)	14.17 (7.64–20.91)	*p*^KW^ = 0.319
IgD^−^CD27^+^ SM [% of CD45^+^CD19^+^], median, IQR	3.28 (1.10–6.38)	1.54 (1.45–3.00)	5.94 (1.21–11.81)	*p*^KW^ = 0.357
IgD^−^CD27^−^ DN [% of CD45^+^CD19^+^], median, IQR	3.19 (1.57–4.17)	2.72 (1.71–3.49)	4.19 (2.18–6.32)	*p*^KW^ = 0.509
IgD^+^CD27^−^ NAIVE [% of CD45^+^CD19^+^], median, IQR	77.67 (65.77–78.76)	74.35 (63.04–75.67)	76.80 (60.37–78.26)	*p*^KW^ = 0.811
CD19^+^CD25^+^ [% of CD45^+^CD19^+^], median, IQR	13.21 (7.57–15.23)	15.85 (13.63–21.38)	17.51 (10.83–21.75)	*p*^KW^ = 0.160
CD19^+^CD25^+^FoxP3^+^ [%of CD45^+^CD19^+^], median, IQR	0.60 (0.20–2.01)	2.21 (0.80–2.76)	1.37 (0.38–1.84)	*p*^KW^ = 0.148
CD19^+^CD25^+^FoxP3^−^ [% of CD45^+^CD19^+^], median, IQR	9.97 (6.90–15.11)	14.97 (10.00–18.14)	16.73 (10.78–19.64)	*p*^KW^ = 0.223

AZS = atopic dermatitis; IQR = interquartile range; KW = Kruskal–Wallis test; SM = switched memory; DN = double negative.

**Table 5:** Subpopulations of B-cell subsets in the group of children with completely controlled, partially controlled, and uncontrolled allergic rhinitis.

Variable	Completely Controlled AR (n = 26)	Partially Controlled AR (n = 8)	Uncontrolled AR (n = 7)	*p* Value
CD45^+^CD19^+^ [% of total]; median, IQR	11.76 (8.37–14.70)	9.02 (7.21–15.17)	11.16 (9.67–16.15)	*p*^KW^ = 0.796
CD38^hi^CD24^hi^ TRANS [% of CD45^+^CD19^+^], median, IQR	9.69 (8.18–15.46)	7.31 (4.12–11.44)	8.32 (5.20–14.54)	*p*^KW^ = 0.198
CD38^hi^CD24^lo^ PLASMABL [% of CD45^+^CD19^+^], median, IQR	0.40 (0.22–0.66)	0.36 (0.21–0.39)	0.35 (0.28–0.44)	*p*^KW^ = 0.865
IgD^+^CD27^+^ NSM_MZ [% of CD45^+^CD19^+^], median, IQR	18.13 (10.87–22.30)	24.96 (16.40–37.30)	19.10 (9.01–26.49)	*p*^KW^ = 0.100
IgD^−^CD27^+^ SM [% of CD45^+^CD19^+^], median, IQR	2.27 (1.02–9.21)	1.82 (0.97–7.65)	3.27 (3.00–4.84)	*p*^KW^ = 0.362
IgD^−^CD27^−^DN [% of CD45^+^CD19^+^], median, IQR	3.77 (1.54–5.31)	2.57 (2.00–3.62)	4.17 (2.27–7.10)	*p*^KW^ = 0.637
IgD^+^CD27^−^ NAÏVE [% of CD45^+^CD19^+^], median, IQR	73.45 (63.04–81.62)	70.90 (54.88–75.61)	66.67 (62.43–85.56)	*p*^KW^ = 0.347
CD19^+^CD25^+^ [% of CD45^+^CD19^+^], median, IQR	14.36 (9.65–18.40)	22.24 (14.25–25.19)	18.18 (6.62–20.72)	*p*^KW^ = 0.070
CD19^+^CD25^+^FoxP3^+^ [% of CD45^+^CD19^+^], median, IQR	1.34 (0.35–2.22)	2.35 (1.65–3.26)	0.64 (0.23–0.97)	*p*^KW^ = 0.043
CD19^+^CD25^+^FoxP3^−^ [% of CD45^+^CD19^+^], median, IQR	13.17 (7.93–16.49)	19.86 (11.70–20.86)	14.12 (6.47–19.21)	*p*^KW^ = 0.116

AR = allergic rhinitis; IQR = interquartile range; KW = Kruskal–Wallis test; NSM_MZ = non-switched-memory marginal zone; SM = switched memory; DN = double negative.

**Table 6:** Antibody panel for flow cytometric analysis of B-cell subsets.

Population	Antibody	Fluorochrome	Cat. No.	Manufacture	Clone
B-cell subsets	CD19	Brilliant Violet 785	363028	BioLegend, San Diego, CA, USA	SJ25C1
	CD24	ECD	B12699	Beckman Coulter, USA	ALB-9
	CD25	PC5	IM2646	Beckman Coulter, USA	B.149.9
	CD27	PC7	B49205	Beckman Coulter, USA	1A4CD27
	CD38	Brilliant Violet 605	356642	BioLegend, USA	HB-7
	CD45	PB	A74763	Beckman Coulter, USA	J33
	IgD	APC	B30655	Beckman Coulter, USA	IA6-2
	FoxP3	R718	566935	BD Bioscience, Milpitas, CA, USA	259D/C7

## Data Availability

The data presented in this study are available on reasonable request from the corresponding author. The data are not publicly available due to ethical restrictions.

## Abbreviations

The following abbreviations are used in this manuscript:

AD	Atopic dermatitis
ACT	Asthma Control Test
AR	Allergic rhinitis
Breg	Regulatory B cell
CRP	C-reactive protein
EDTA	Ethylenediaminetetraacetic acid
FoxP3	Forkhead box P3
GINA	Global Initiative for Asthma
IgD	Immunoglobulin D
IgE	Immunoglobulin E
IL-10	Interleukin 10
IL-35	Interleukin 35
PBMCs	Peripheral blood mononuclear cells
SCORAD	SCORing Atopic Dermatitis
TGF-beta	Transforming growth factor beta
Th1	T helper type 1
Th2	T helper type 2
Th17	T helper type 17
VAS	Visual analogue scale
WBC	White blood cel count
